# Impact of Scapular Repositioning Using a Scapular Belt for Axioscapular Muscle Imbalance in Patients With a Non-specific Neck Pain: A Case Report

**DOI:** 10.7759/cureus.28126

**Published:** 2022-08-18

**Authors:** Pooja Chaurasia, Shrikant Mhase, Manibhadra Panda, Sabih Khan, Vikas Bedre, Wruchika Nagrale

**Affiliations:** 1 Rehabilitation, Mahatma Gandhi Mission (MGM) School of Physiotherapy, Aurangabad, IND; 2 Community Physiotherapy, Mahatma Gandhi Mission (MGM) School of Physiotherapy, Aurangabad, IND; 3 Cardiorespiratory Physiotherapy, Mahatma Gandhi Mission (MGM) School of Physiotherapy, Aurangabad, IND; 4 Research, NKP Salve Institute of Medical Sciences and Research Centre, Nagpur, IND

**Keywords:** axioscapular muscle imbalance, poor work ergonomics, forward head posture, scapular dyskinesis, scapular belt, scapular repositioning, chronic neck pain

## Abstract

A common cervical spine condition that frequently interferes with a patient's daily activities is chronic neck pain. The axioscapular muscle imbalance that results from increased middle trapezius activity in patients with non-specific chronic neck pain affects cervical spine stability and contributes to pain. A 67-year-old male, who was a retired office secretary, experienced neck pain for two years. In the last four months, his discomfort worsened, impairing his ability to function and degrading his quality of life. For the axioscapular muscle imbalance, scapular repositioning with a scapular belt was used to alleviate muscular imbalances and help with posture correction. The results were calculated using the Numerical Pain Rating Scale (NPRS) and the Neck Disability Index (NDI), which revealed significant changes in pain intensity from 9 to 4, and disability scores shifted from 48% to 20% between the pre and post-treatment sessions, respectively. In order to decrease neck pain, this case report investigates the impact of scapular realignment using a scapular belt in the treatment of non-specific chronic neck discomfort.

## Introduction

Chronic pain is a significant healthcare issue affecting 17-27% of people worldwide. Chronic neck pain and discomfort are a frequent cause of impairment in adults (ages 15 to 74), out of which women suffer from neck pain more than men [1]. The most frequent causes of neck pain are poor ergonomics and holding an abnormal posture for an extended period of time. These conditions can result in myofascial pain syndrome, restricted cervical mobility, and cervical spine misalignment, which negatively impact a person's quality of life [2].

The cervical spine is susceptible to higher mechanical stresses and compressive forces when people spend a lot of time on computers, e.g. office secretaries with their heads protracted forward and the scapulae stretched and twisted downward. Axioscapular muscle imbalance brought on by such alterations in the cervical spine alignment results in stiffness and pain in the neck by changing the mobility and orientation of the scapulae and thoracic vertebrae [3,4]. The evaluations of the scapular position indicate the corrective application of the scapular belt. The scapula was assessed for superior or inferior rotation, abducted, rotated superiorly, and protracted rotated inferiorly. The positional alterations determine the position of the scapular belt for scapular repositioning, which involves applying overpressure to the scapular muscles in a figure-of-eight pattern to retract the shoulder backward in order to address the faulty scapular position that can cause neck pain [5]. In many people, overuse of the shoulder changes the scapula's resting position, which causes scapular dyskinesis and, ultimately, neck pain. The current case report aimed at assessing the benefits of the scapular belt in establishing optimal scapular alignment, which should be a key goal of treatment plans for chronic neck pain.

## Case presentation

A 67-year-old male, retired office secretary, walked in with the chief complaint of neck pain for the past two years, which was aggravated for four months and was continuous in nature. The cervical extension was completely restricted and even the slight movement was aggravating the neck pain. On observation, the patient showed postural deviations that involved the forward head posture and protracted shoulders. The semispinalis capitis and splenius capitis exhibited trigger points and grade 1 tenderness over the spinous process of the cervical spine on palpation. On assessment, the cervical extension range of motion (ROM) was limited and painful. Resisted isometrics for cervical extensors were weak and painful. The diagnosis of non-specific neck pain was determined based on the Numeric Pain Rating Scale (NPRS) and the Neck Disability Index (NDI). The patient's pain level was 9 on the NPRS during activity, and an NDI score of 48% was obtained.

**Figure 1:**
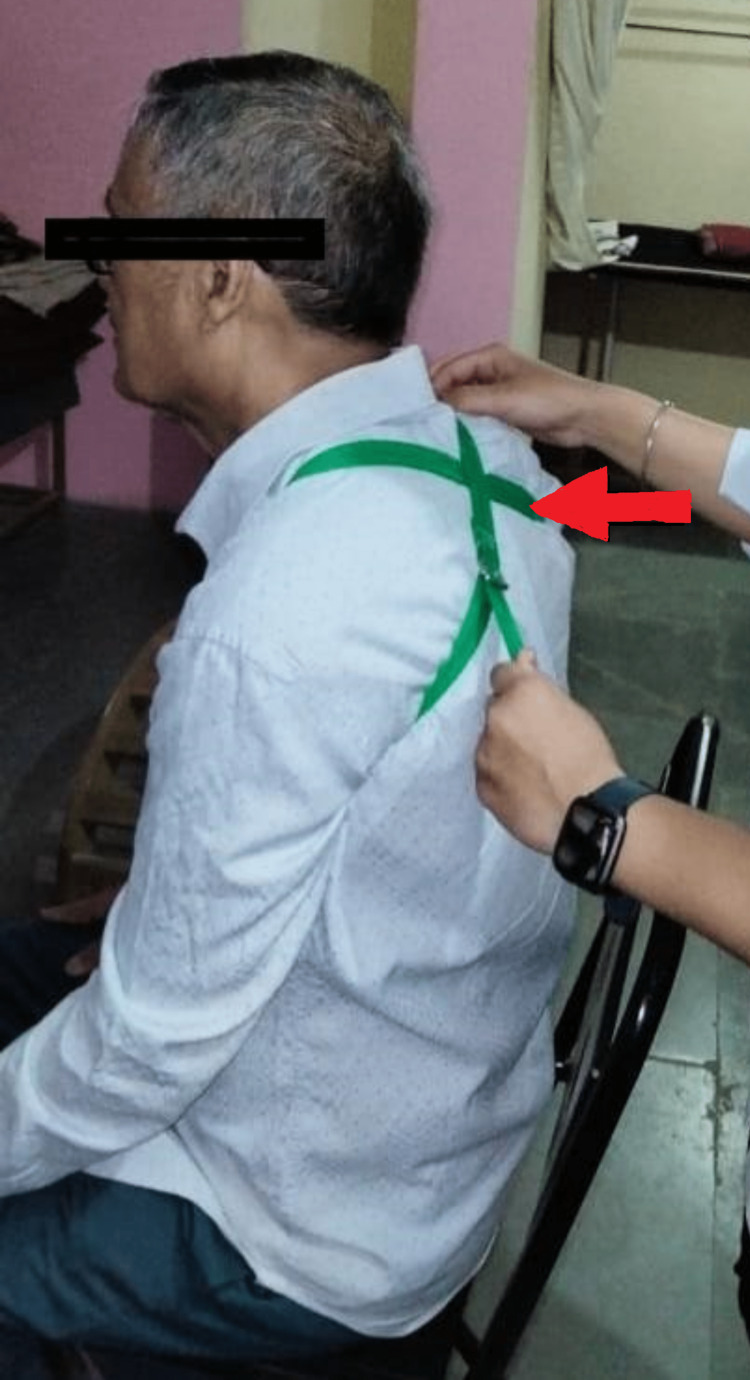
Scapular realignment using a scapular belt

The scapula was repositioned by wrapping a scapular belt over the shoulders in a figure-of-eight eight pattern, pulling the shoulders back while exerting excessive pressure on the muscles in the rear of the scapula to rectify its forward protraction. While the therapist stands behind the patient, the patient is asked to sit comfortably on a chair. The fixation end of the belt is placed over the posterior aspect of the scapula, and the free end is rounded around the right shoulder to the left shoulder in a figure-of-eight pattern. Pressure is then applied posteriorly to the scapula in order to retract it. Force is adjusted as per the compliance of the patient in order to reposition the scapula. The patient was advised to apply the belt three days a week for 20 minutes for three weeks (Figure 1) [4].

After three sessions of scapular realignment using a scapular belt, tucked chin and neutral shoulders were observed on observation. The pain intensity decreased significantly from 9 to 5 on the NPRS with improved scapular alignment. The positional alterations of the scapula before the treatment marked superior rotation, abduction, and protraction, which after the treatment was inferior rotation, adduction, and retraction to the neutral position. The cervical extension showed an improvement of 0-50 degrees. The distance between the medial border of the scapula and the spinous process was measured at the T4 level. Before the treatment, the distance was 18 centimeters (cm) on each side, and after the treatment, it was reduced to 8 cm on each side. Additionally, the NDI score improved from 48% to 20% as shown in Table 1.

**Table 1:** Pre and post-treatment improvement in NPRS, cervical extension, medial border of the scapula and spinous process distance, and NDI using a scapular belt

Outcome Measures	Pre-treatment	Post-treatment
Numerical Pain Rating Scale (NPRS)	9	5
Cervical extension	0-10°	0-50°
Distance between the medial border of the scapula and the spinous process at the T4 level	18 centimeters (cm)	8 cm
Neck Disability Index (NDI)	48%	20%

The patient was co-operative and adhered well to the treatment. There were no adverse and unanticipated events.

## Discussion

Axioscapular muscle dysfunction plays a major role in neck pain. It is greatly affected by the posture adopted by the individual and the alignment of the spine and shoulder. Changes in the cervical spine's alignment, such as a forward head posture or protracted shoulders, have an impact on the movement and positioning of the scapulae and thoracic vertebrae. This imbalance in the axioscapular muscles causes neck pain [6,7].

People with axioscapular muscle imbalance exhibit higher middle trapezius activity than lower trapezius. The rationale for this is that the scapula and the neck have similar muscle attachments [8,9].

Barbari et al. devised the use of scapular repositioning intervention in the axioscapular muscles' activity, which aids in proper alignment by adjusting the scapula's position and correcting posture by releasing the tension in overly tight muscles. It also helps decrease the imbalance between the axioscapular muscles, which has an impact on the neck muscles and eases pain as assessed clinically [10].

This case report is distinct from earlier research investigations since it aims to demonstrate scapular repositioning by employing a scapular belt for persistent non-specific neck pain.

## Conclusions

This case report illustrated the benefits of the scapular belt for the management of non-specific chronic neck pain, and it suggests that scapular realignment with a scapular belt may be helpful for people with chronic non-specific neck pain in order to hasten the outcomes of physical treatment as people with axioscapular muscle imbalance exhibit higher middle trapezius activity than lower trapezius.
